# A study to translate and validate the Thai version of the Victoria Respiratory Congestion Scale

**DOI:** 10.1186/s12904-022-01043-x

**Published:** 2022-08-27

**Authors:** Tuangporn Tantiwatniyom, Kittiphon Nagaviroj

**Affiliations:** Department of Family Medicine, Faculty of Medicine, Ramathibodi Hospital, Mahidol University, Bangkok, Thailand

**Keywords:** VRCS, Death rattles, Anticholinergics, Secretion

## Abstract

**Purpose:**

Few clinical tools are available to objectively evaluate death rattles in palliative care. The Victoria Respiratory Congestion Scale (VRCS) was adapted from the Back's scale, which has been widely utilized in research and clinical practice. The VRCS will be translated into Thai and research will be conducted to determine its validity and reliability in assessing death rattles in palliative care.

**Methods:**

Two qualified language specialists converted the original tool into Thai and then back to English. Between September 2021 and January 2022, a cross-sectional study was undertaken at a palliative care unit at Ramathibodi Hospital to determine the Thai VRCS's validity and reliability. Two evaluators independently assessed the volume of secretion noises using the Thai VRCS. The criterion-related validity of VRCS was determined by calculating the correlation between the sound level obtained with a standard sound meter and the VRSC scores using Spearman's correlation coefficient method. To assess inter-rater reliability and agreement measurement on ratings, we utilized a two-way random-effects model with Cohen's weighted kappa agreement.

**Results:**

Forty patients enrolled in this study with a mean age of 75.3 years. Fifty-five percent had a cancer diagnosis. Spearman's rho correlation coefficient was found to be 0.8822, *p* < 0.05, indicating a highly significant link. The interrater reliability analysis revealed that the interrater agreement was 95% and the Cohen's weighted kappa agreement was 0.92, indicating near-perfect agreement.

**Conclusions:**

Thai VRCS demonstrated excellent criteria-related validity and interrater reliability. Using the Thai VRCS to assess adult palliative care patients' death rattles was recommended.

## Background

Death rattles is one of the most common symptoms in end-of-life patients. Previous research estimated that the prevalence of death rattles to be between 12–92 percent [1]. Although the exact etiology of this symptom is unknown, many assume it is caused by the patient's inability to cough or swallow oral and bronchial secretions [2]. As a result, secretions from the airways accumulate in the throat. When the breathed and exhaled air passes through the liquid, a screaming sound is produced. Although the effect on the patient itself is unclear, most experts believe that patients may not be aware of the symptom because of decreased level of consciousness during the last days [1]. Nevertheless, this symptom may cause significant anxiety and concerns among family, caregivers, healthcare professionals, or even the patient nearby. Additionally, it caused families and caregivers distress as they witnessed their loved ones' suffering, which was possibly interpreted by the family as “choking to death”. A previous study discovered that nurses caring for patients with death rattle reported that the patient's relatives felt the patient was in a lot of suffering. It's like the patient is "gagging" or "drowning," while some relatives think it's useful as a warning sign that the patient will die soon [1]. However, there is no clear evidence that death rattle is associated with respiratory distress in this group of patients.

There are still few clinical tools available to evaluate death rattles in palliative care patients. The Back's scale [3] is a well-known clinical tool for assessing the severity of respiratory congestion or death rattle. It was originally developed for use in a study comparing the efficacy of subcutaneous hyoscine hydrobromide and glycopyrrolate in reducing death rattles in patients entering the final stages of life in a specialist palliative care unit. Scale 0 is inaudible, scale 1 is heard close to the patient, scale 2 is clearly heard at the end of the bed in a quiet room, and scale 3 is clearly heard at approximately 20 feet (9.5 m) or at the door in a quiet room. This tool had been used in six previous studies, four of which reported the percentage of patients in each grade. Scale 1 was received by 6–17 percent, scale 2 by 19–26 percent, and scale 3 by 5–11 percent. Even though the authors evaluated the face validity of the Back's scale. However, no data on its validity or reliability were published [4].

The Victoria Hospice Society of Canada then modified and developed the Back's scale into the Victoria Respiratory Congestion Scale (VRCS), which was first published in the Medical Care of the Dying Textbook (2006) and is referenced on the Victoria Hospice Society Web Site [5]. This tool classified the level of secretion sound into four levels: 0 = no congestion, 1 = audible at 12 inches (30 cm) from the patient's chest but not further, 2 = audible at the end of the bed but not further, and 3 = audible at the room's doorway. Compared to the Back's scale, we discovered that the VRCS provided more specific and clear instructions on how to use the tool, such as clarifying the distance between the measuring point and the patient's chest for score 1, indicating that the distance from the bed is based on an approximate single room, recommending reducing room noise as much as possible during the assessment, and recommending repeated measurements. We contacted the Victoria Hospice Society to obtain permission to use the Victoria Respiratory Congestion Scale (VRCS) and to inquire about the tool's validity and reliability. It was discovered that the VRCS had never been tested for validity and reliability. As a result, we are interested in translating the VRCS assessment into Thai and conducting a study to test its validity and reliability in assessing the loudness of death rattles in palliative care patients nearing the end of their lives.

## Methods

We initially requested permission from the Victoria Hospice Society to translate the original tool into Thai. The Victoria Respiratory Congestion Scale (VRCS) was translated into Thai and then back to English by two certified language experts. Three palliative care specialists checked the Thai version of VRCS for accuracy. A cross-sectional study was then conducted in a specialized palliative care unit at Ramathibodi Hospital in Bangkok between September 2021 and January 2022 to determine the validity and reliability of the Thai VRCS.

### Ethics

The Human Research Ethics Committee approved this research project, Faculty of Medicine, Ramathibodi Hospital, Mahidol University Project No. COAL. MURA2021/712, on August 23rd, 2021. All methods were carried out in accordance with the approved study protocol under the Declaration of Helsinki. Participants were informed of the purpose and procedures of the study prior to the start of the study and had the right to withdraw at any time. Informed written consent was obtained from all participants prior to participation.

### Inclusion and exclusion criteria

Patients over the age of 18 with a prognosis of less than a week or a high likelihood of death within 48–72 h are eligible. The patient's prognosis was determined by the presence of more than two common signs and symptoms observed in the last few days of life, such as a decreased level of consciousness or increased sleepiness. confusion and restlessness, difficulty swallowing, inability to eat or drink, death rattles, inability to close eyelids, air hunger, Cheyne stoke breathing or intermittent apnea, low blood pressure not associated with dehydration, pulselessness of radial artery, and low urine output [6, 7]. If patients withdrew from the study, they were excluded from the analysis.

### Data collection

The data for the study was gathered using a standardized data record form. The data set was comprised of the patient’s profile (age, gender, marital status, health insurance) and disease status (principal diagnosis, comorbidities, metastases). After receiving permission from the patient or relatives to participate in the study, two assessors comprised of palliative care physicians and nurses working in the palliative care unit used the Thai version of VRCS to assess the volume of secretion sounds. To blind the assessment score, the two assessors separately wrote down the score level on the data record form and place it in a sealed envelope. Each assessor was unaware of the other assessor's evaluation score.

#### Sound level metering

During the same period that the two assessors rated the VCRS, the researcher measured the sound level with a standard sound level meter. The 3MTM SoundProTM SE and DL Series Sound Level Meters meet the IEC 61,672 class 2 standard, as recommended by the Speech Sound Level Measurement Guidelines [8], and measure sound level in decibels. Before each measurement, the sound meter was calibrated with an acoustic calibrator. Each measurement lasted one minute, and the researcher recorded the average or equivalent sound level (mean LAeq) as well as the maximum sound level in decibels. To avoid unwanted noise during the measurement, we used a sliding wall between the beds, all medical devices were muted, and all medical staff was asked to remain silent. The measurement was repeated twice, five minutes apart. The correlation with VCRS scores was determined using the average sound level of the two measurements. The two VRCS assessors will not know the measurement results, and the researcher will not know the two assessors' VCRS scores.

### Statistical analysis

The characteristics of the participants were presented in terms of frequency and percentage of the categorical data and mean, with standard deviation for continuous data, if the data had a normal distribution. If that were not the case, the median with range was applied. The sample size was determined by using the Sample Size Charts for Spearman and Kendall Coefficients [9] by setting power 80%, significance level (α) = 0.05, and alternative value (ρs1) = 0.4 (moderate correlation). A sample size of 40 people was used to calculate the proportion of score 0: 1: 2: 3 based on the prevalence of each score from the systematic review [1], which was approximately 23: 5: 9: 3 people in each group. The criterion-related validity of VRCS was calculated using Spearman's correlation coefficient statistical method from the correlation between the sound level in decibels and the VRSC scores. The criteria used to determine the degree of Pearson's and Spearman's correlation coefficients were based on Chan et al. guidelines [10, 11]. If the correlation coefficient is 1, it is highly correlated (Perfect); if the correlation coefficient is between 0.80–0.99, it is very strong; if the correlation coefficient is between 0.60–0.79, it is moderate. A correlation coefficient between 0.30–0.59 indicates a fair correlation, a correlation coefficient between 0.10–0.29 indicates a very low correlation, and a correlation coefficient of 0 indicates no correlation.

The two-way random-effects model with Cohen's weighted kappa agreement was used to examine interrater reliability and agreement measurement on ratings. The Landis and Koch guideline was used to determine the level of correlation of the kappa statistics [12, 13]. If the kappa value is between 0.81–1.00, it is considered almost perfect; if it is between 0.61. -0.80, the consistency is substantial; kappa between 0.41- 0.60, moderate; kappa between 0.21- 0.40, fair; kappa between 0.00- 0.20, slight; and kappa less than 0, there is no correlation (poor).

The STATA version 18.0 program was used to analyze the statistical data, and the level of significance was set at 0.05.

## Results

The study included forty palliative care patients who were nearing the end of their lives. The age range of the 40 patients ranged from 43 to 96 years, with a mean age of 75.3 years. Fifty-seven point five percent of those polled were female, and 55 percent had cancer. The non-cancer diagnoses included pneumonia (32.5%), septicemia (15%), end-stage renal disease (10%), cerebrovascular disease (7.5%), coronary artery disease (5%), heart failure (2.5%) and necrotizing fasciitis (2.5%). The most common types of cancer among patients with a cancer diagnosis were hepatobiliary cancer (22.7%), breast cancer (18.2%), colorectal cancer (18.2%), and cancer of the urinary tract (18.2%). The most common sites of metastasis were the lung (50%) and intraperitoneal (31.8%) metastases, as well as bone metastases (18.2%). Table 1 shows the characteristics of study participants.

**Table 1:** Characteristics of study participants (*N* = 40)

Characteristics	Number (%)
Gender	
- Male	17 (42.5)
- Female	23 (57.5)
Age group (years)	
- ≤ 60	6 (15)
- 61–70	9 (22.5)
- 71–80	8 (20)
- 81–90	14 (35)
- ≥ 91	3 (7.5)
Major diagnosis	
- Pneumonia	13 (32.5)
- Cancer	10 (25)
- Septicemia	6 (15)
- End-stage renal disease (ESRD)	4 (10)
- Cerebrovascular disease	3 (7.5)
- Coronary artery disease	2 (5)
- Heart failure	1 (2.5)
- Necrotizing fasciitis	1 (2.5)
Co-morbidities	
- Hypertension	20 (50)
- Cardiac diseases	14 (35)
- Chronic kidney diseases	8 (20)
- Dyslipidemia	8 (20)
- Diabetes mellitus	7 (17.5)
Type of malignancies (*N* = 22)	
- Hepatobiliary cancer	5 (22.7)
- Breast cancer	4 (18.2)
- Colorectal cancer	4 (18.2)
- Cancer of the urinary tract	4 (18.2)
- Lung cancer	1 (4.6)
- Esophageal and gastric cancer	1 (4.6)
- Ovarian and endometrial cancer	1 (4.6)
- Skin cancer	1 (4.6)
- Cancer of parotid gland	1 (4.6)
Metastases (*N* = 22)	
- Lung metastases	11 (50)
- Intraperitoneal metastases	7 (31.8)
- Bone metastases	4 (18.2)
- Pleural metastases	3 (13.6)
- Lymph node metastases	2 (9.1)

### VRCS scores and sound level in decibels

There were 23 patients who received VRCS = 0, 5 patients who received VRCS = 1, 9 patients who received VRCS = 2, and 3 patients who received VRCS = 3. (Table 2). Table 3 shows the mean average or equivalent sound level (mean LAeq) for each VRCS score.

**Table 2:** Number of patients in each VRCS score (*N* = 40)

VRCS score	Number (percent)
0	23 (57.5)
1	5 (12.5)
2	9 (22.5)
3	3 (7.5)

**Table 3:** The mean average or equivalent sound level (mean LAeq) in each VRCS score (*N* = 40)

VRCS score	Number of patients	Mean LAeq (SD)	Minimum LAeq	Maximum LAeq
0	23	49.3 (1.1)	47	51
1	5	51.6 (0.6)	50.9	52.6
2	9	54 (1.5)	51.7	56.2
3	3	56.9 (1.5)	55.5	58.4

**Fig. 1:**
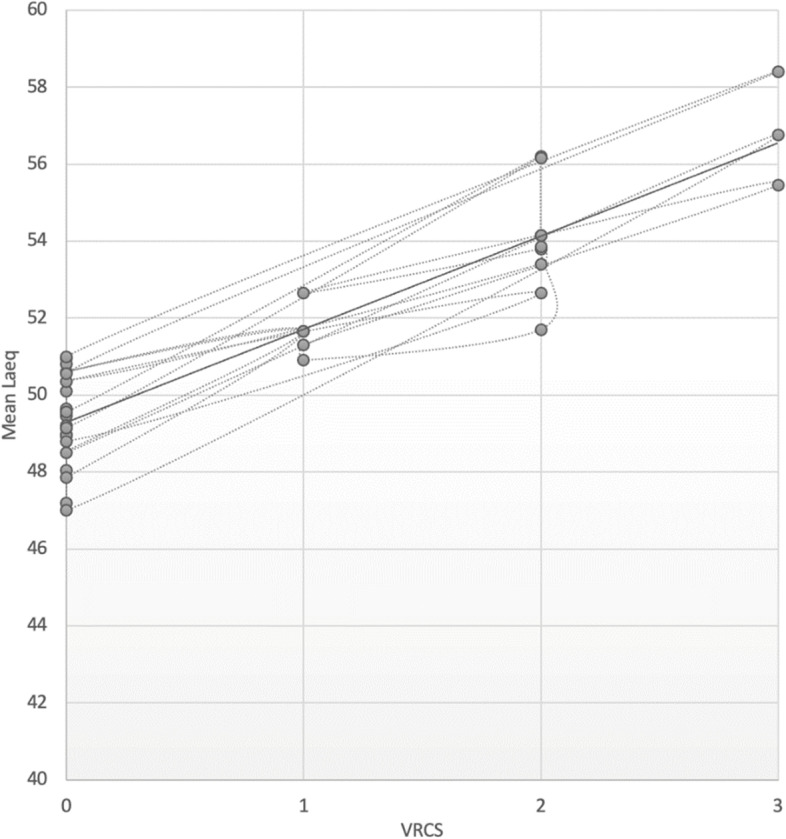
The scatter plot depicts the linear correlation between VRCS score and average sound level in decibels

**Fig. 2:**
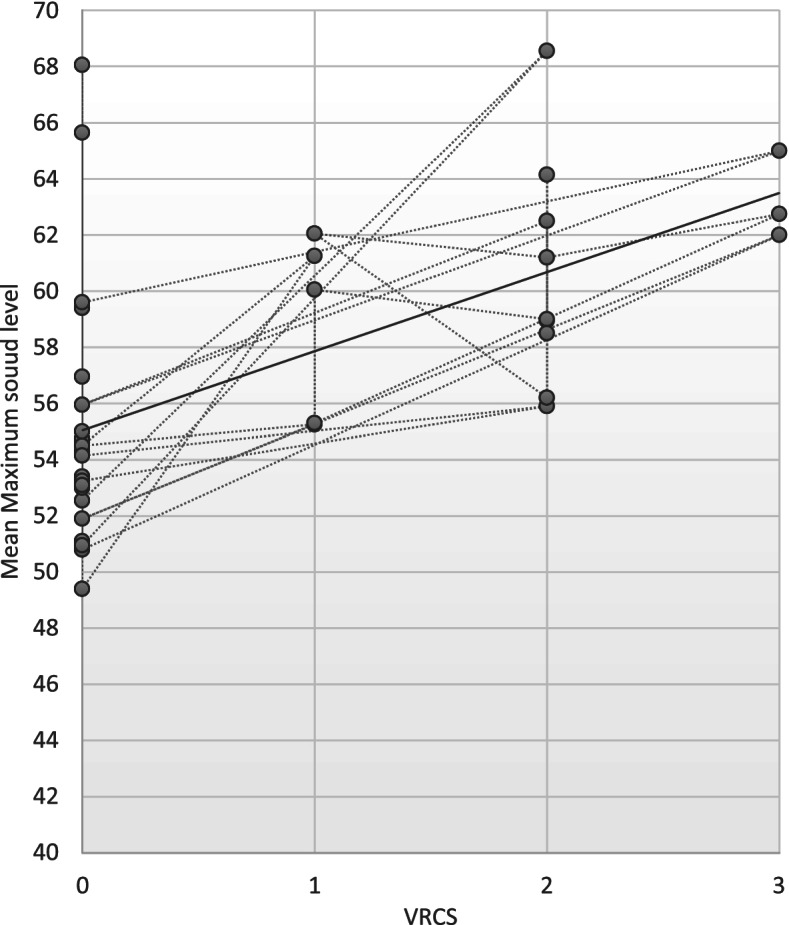
The scatter plot depicts the linear correlation between VRCS score and maximum sound level in decibels

Spearman’s correlation coefficient was used to analyze the correlation coefficient between Thai VRCS scores and sound level measured with a standard sound meter. Spearman's rho correlation coefficient was found to be 0.8822, *p* < 0.05. The level of correlation could be interpreted as a very strong correlation with statistical significance, according to the guidelines of Chan et al [10, 11]. The scatter plot of the correlation between the Thai version of the VRCS score and the mean sound level in decibels was also found to be linearly and positively correlated, as shown in Fig. 1. A sensitivity analysis was also performed to determine the correlation between the maximum sound level and the Thai VRCS scores, which revealed a Spearman's rho correlation coefficient of 0.6422, p 0.05. The level of correlation could be interpreted as moderate with statistical significance. Figure 2 depicts a scatter plot of the correlation between the Thai version of the VRCS score and the maximum sound level in decibels.

### Interrater reliability of VRCS score

To identify the agreement measurement on ratings between two assessors, the interrater reliability was calculated using a two-way random-effects model. The analysis of the data showed that the interrater agreement was 95% and the Cohen's weighted kappa agreement was 0.92, which was considered to be almost perfect agreement according to Landis and Koch's guideline [12, 13]. (See Table 4).

**Table 4:** Demonstrate the level of agreement and Cohen’s weighted kappa agreement

Test	Agreement	Expected agreement	Kappa	Std.error	Z	Prob > Z
Thai VRCS	95.00%	39.50%	0.9174	0.1010	9.09	0.0000

## Discussion

To the best of our knowledge, this is the first study to show the criterion-related validity and reliability of the Victoria Respiratory Congestion Scale, a clinical tool used to objectively assess death rattles in palliative care patients. The tool has been widely used in past research and clinical practice. The Thai version of the Victoria Respiratory Congestion Scale was found to be highly correlated with the sound level measured by a standard sound meter in this study. Criterion-related validity was very strong and statistically significant (Spearman’s rho = 0.8822, *p* < 0.05), and the interrater reliability was at a nearly perfect level and statistically significant (Cohen's weighted kappa agreement = 0.9174, *p* < 0.05). The findings of our study are consistent with the findings of Downing M.'s previous study, which demonstrated the concurrent validity of the original VRCS (*p* < 0.001) [14]. Our findings also revealed that Thai VRCS have higher interrater reliability than one previous study, which found only a moderate level of reliability (k = 0.53, *p* < 0.001) [14]. However, we were unable to locate the original published data from the earlier study in order to investigate the differences further.

The difference in interrater reliability between the two studies could be explained by the distance between the patient's bed and the room's doorway. If the assessor can hear the death rattles at the doorway, the VRCS score is 3 points in the original version. The distance may vary depending on the size of the room and affect the evaluation of VRCS scores. We contacted the Victoria Hospice Society to clarify the issue because there was no specific recommendation mentioned in the original tool. Their VRCS = 3 recommendation was to hear the death rattles at a distance of 12–16 feet from the patient's bed. As a result, we decided to use the standard distance of 4 m from the end of the bed as our reference standard in our study. The standardized measurement distance may aid in increasing the tool's interrater reliability.

It is also worth noting that the VRCS and another commonly used tool, the Back's scale, have some differences. A score of 1 on the Back's scale indicates that the death rattle can be heard close to the patient. The VRCS score of 1 indicates that the death rattle is audible at 12 inches (30 cm) from the patient's chest but not further away. There was also a difference in how those tools defined the distance to the doorway. According to the Back's scale, a score of 3 indicates that the death rattle is clearly heard at approximately 20 feet (9.5 m) or at the door in a quiet room. The VRCS score of 3 indicates that the death rattle can be heard from the room's doorway (12–16 feet), based on the approximate size of a single room. Furthermore, the Back's scale was designed to be used in a quiet environment. The VRCS, on the other hand, can be used when ambient noise is kept to a minimum during the assessment. This is closer to actual palliative care settings. As a result, we believed the VRCS provided more specific instructions and was convenient to use in palliative care patients.

### Limitations

There were some limitations to our study that should be mentioned. Although the proportion of patients in each death rattle score from our study is comparable to the findings of the previous systematic review [1]. The fact that more than half of the study's participants obtained a VRCS score of 0 may have affected the study's results and overestimated the reliability of the tool. Despite the fact that we followed the standard sound measurement protocol, including the use of an IEC 61,672 class 2 sound meter and measuring techniques recommended by the Speech Sound Level Measurement Guidelines [8]. Some difficulties included attempting to avoid uncontrollable background noises in the palliative care unit, such as snoring or yelling from another confused patient. This may have an impact on the precision of noise level measurements as well as VRCS assessments. However, these noises are very likely to occur in real clinical practice and are unavoidable. As a result, we believe that these factors reflect their practical application of this tool and should not have an impact on the validity and reliability of our study. We also did not conduct the definite hearing tests required by the assessors who rated the VRCS score. In the case of the assessor's hearing impairment, this could have an impact on VRCS accuracy.

Furthermore, this study was only conducted in a specialized palliative care unit, which was generally calmer than general medical wards. If the tools are used in other clinical settings, such as intensive care units or emergency rooms, where there may be more disturbing noise, the results may differ. This study included palliative care patients aged 18 and up, with the majority of participants being elderly. As a result, its use in pediatric populations may be limited and warrants further investigation.

## Conclusion

When compared to the sound level measured by a standard sound meter, Thai VRCS had very strong criteria-related validity (Spearman's rho = 0.8822, *p* < 0.05), and the almost perfect level of the interrater reliability (Cohen's weighted kappa agreement = 0.9174, *p* < 0.05). The Thai VRCS was recommended as the standard assessment tool for death rattles in adult palliative care patients.

## Data Availability

All data sets on which the conclusions of the paper are based are available upon request to the corresponding author.
